# Interaction between genetic predisposition, smoking, and dementia risk: a population-based cohort study

**DOI:** 10.1038/s41598-021-92304-x

**Published:** 2021-06-21

**Authors:** Na Zhang, Janice M. Ranson, Zhi-Jie Zheng, Eilis Hannon, Zhenwei Zhou, Xuejun Kong, David J. Llewellyn, Daniel A. King, Jie Huang

**Affiliations:** 1 Department of Global Health, Peking University School of Public Health, Beijing, China; 2 College of Medicine and Health, University of Exeter, Exeter, UK; 3 Department of Biostatistics, Boston University, Boston, MA USA; 4 Department of Psychiatry, Beth Israel Deaconess Medical Center, Boston, MA USA; 5 Martinos Center, Massachusetts General Hospital, Charlestown, MA USA; 6 Alan Turing Institute, London, UK; 7 Department of Medicine, Stanford University School of Medicine, Stanford, CA USA; 8 School of Public Health, Peking University Health Science Center, Haidian District, No. 38 Xueyuan Road, Beijing, 100191 P. R. China; 9 Institute for Global Health and Development, Peking University, Beijing, China

**Keywords:** Dementia, Risk factors, Neurodevelopmental disorders, Geriatrics, Public health

## Abstract

We evaluated whether the association between cigarette smoking and dementia risk is modified by genetic predisposition including apolipoprotein E (APOE) genotype and polygenic risk (excluding the APOE region). We included 193,198 UK Biobank participants aged 60–73 years without dementia at baseline. Of non-APOE-ε4 carriers, 0.89% (95% CI 0.73–1.08%) current smokers developed dementia compared with 0.49% (95% CI 0.44–0.55%) of never smokers (adjusted HR 1.78; 95% CI 1.39–2.29). In contrast, of one APOE-ε4 allele carriers, 1.69% (95% CI 1.31–2.12%) current smokers developed dementia compared with 1.40% (95% CI 1.25–1.55%) of never smokers (adjusted HR 1.06; 95% CI 0.77–1.45); of two APOE-ε4 alleles carriers, 4.90% (95% CI 2.92–7.61%) current smokers developed dementia compared with 3.87% (95% CI 3.11–4.74%) of never smokers (adjusted HR 0.94; 95% CI 0.49–1.79). Of participants with high polygenic risk, 1.77% (95% CI 1.35–2.27%) current smokers developed dementia compared with 1.05% (95% CI 0.91–1.21%) of never smokers (adjusted HR 1.63; 95% CI 1.16–2.28). A significant interaction was found between APOE genotype and smoking status (*P* = 0.002) while no significant interaction was identified between polygenic risk and smoking status (*P* = 0.25). APOE genotype but not polygenic risk modified the effect of smoking on dementia risk.

## Introduction

Dementia is one of the major causes of disability and dependency in later life^1^. Both genetic and lifestyle factors contribute to the development of dementia^2,3^. Apolipoprotein E (APOE) gene polymorphism is strongly associated with late-onset Alzheimer Disease (AD)^4,5^. It also plays a role in risk for vascular dementia^6^, Lewy body dementia^7,8^ and frontotemporal dementia^9^. There are three APOE isoforms, APOE2, APOE3, APOE4, encoded by 3 common alleles, ε2, ε3 and ε4. Compared to the reference ε3 allele, ε2 is protective against AD, while ε4 is the largest known genetic risk factor of late-onset sporadic AD in a variety of ethnic groups^10^. In European populations, a single ε4 allele increases an individual’s risk for AD by three-fold^11^, while two ε4 alleles increase the rate by nearly 30-fold by 75 years of age. APOE ε4 carriers are also more likely to develop other types of dementia^9,12–14^. A polygenic risk score (PRS) indicates cumulative genetic propensity using multiple risk alleles identified by genome-wide association studies (GWAS)^15^. Individuals with higher PRS are more likely to develop dementia^16,17^, and may also have an earlier onset of the disease^18^.

Compelling evidence suggests that tobacco smoking is associated with a higher risk of dementia^14,19–21^. In addition to an independent effect, smoking may interact with genetic risk factors for dementia. However, prior findings have been inconsistent. Evidence from the population-based Rotterdam study suggested that smoking was only associated with dementia risk in ε4 allele non-carriers^22,23^ whereas a population-based study in Finland found that smoking increased risk of dementia among ε4 allele carriers^24^. Furthermore, no studies to our knowledge have examined the gene-smoking interaction on dementia risk at a genome-wide level. Here, we used prospective data from a large population-based study to examine the interaction between genetic predisposition including APOE genotype and polygenic risk and smoking in contributing to the risk of dementia. It aims to clarify whether dementia risk reduction strategies incorporating smoking cessation are likely to be effective regardless of genotype, or conversely are only likely to be effective in targeted groups.

## Methods

### Study design, data sources and participants

We conducted a population-based retrospective cohort study using UK Biobank data. The UK Biobank (UKB) cohort consists of over 500,000 adults aged 37–73 years who were recruited from 22 research centers across the UK between 2006 and 2010^25^. Our analytic sample was restricted to individuals with White British ancestry because APOE ε4 allele frequencies vary between different populations^26^ and the polygenic risk score for Alzheimer disease was based upon GWAS results from White participants. Our sample was also limited to those at least 60 years old at baseline because our study was focused on the prediction of sporadic late-onset dementia. Participants younger than 60 years (n = 285,022), without ethnic background information (n = 1182) or not White British (n = 17,400), with missing APOE genotype information (n = 5324) or ε1 allele (n = 25), with unmatched self-reported sex and genetic sex (n = 121) and excessive relatives (n = 88), and with dementia at baseline or prior to recruitment (n = 145) were excluded (Supplementary Fig. S1).

### Ethics declarations

This study was conducted according to the Declaration of Helsinki. The generic ethical approval was obtained by UK Biobank from the NHS National Research Ethics Service (approval letter dated June 17th 2011, Ref 11/NW/0382). All participants provided written informed consent to participate in the UK Biobank.

### APOE haplotype

We first identified each subject’s APOE haplotype based on pre-phasing the whole chromosome 19, using the EAGLE software tool^27^. We characterized the haplotype by race, by ethnicity, and by other demographic parameters. We further confirmed the inferred APOE haplotype in 48,855 samples with exome sequencing data. We excluded those predicted with ε1 haplotypes and generated a factor variable with six possible combinations of ε2 to ε4, namely, ε2ε2, ε2ε3, ε2ε4, ε3ε3, ε3ε4, ε4ε4. The six APOE genotypes were classified into three categories: zero ε4 allele (ε2ε2, ε2ε3, ε3ε3), one ε4 allele (ε3ε4, ε4ε4), and two ε4 alleles (ε4ε4) carriers.

### Polygenic risk score

A polygenic risk score (PRS) was calculated using the same methods documented in a previous study^16^. In brief, 249,273 single-nucleotide polymorphisms (SNPs) (excluding the APOE gene) with a *P* value less than 0.5 for association with Alzheimer disease (AD) were used to compute the PRS. The number of risk alleles carried at each SNP were weighted by its effect size in GWAS for AD^28^, then summed and z-standardized^16^. In the present study, the PRS was divided into quintiles and then categorized into low, middle, and high polygenic risk according to lowest quintile, 2–4 quintiles and highest quintile, respectively.

### Dementia diagnosis

Dementia was identified using algorithmically-defined dementia outcomes provided by UKB. It includes primary care information with linked data from hospital admissions and death registries^29^. The hospital and mortality data are coded using the International Classification of Disease version 10 (ICD-10). The algorithms were developed to identify individuals with ICD-10 codes for any cause of dementia and the subtypes including Alzheimer’s disease, vascular dementia and frontotemporal dementia.

### Tobacco smoking

Smoking status was assessed using a touchscreen questionnaire, and defined as (1) never smokers, if individuals specified that they either never smoked tobacco, or just tried once or twice in the past but less than 100 cigarettes over their lifetime; (2) former smokers, if they smoked on most or all days, or occasionally, or just tried once or twice in the past with more than 100 cigarettes over their lifetime; (3) current smokers, if they smoked on most or all days, or occasionally at present.

### Covariates

Covariates measured at the initial assessment visit were incorporated in the analyses as potential confounders. Sociodemographic variables comprised age, sex, educational attainment and deprivation quintiles. Educational attainment was grouped into two categories based on a previous study^30^. Townsend deprivation score^31^ was categorized into least (lowest quintile), intermediate (quintiles 2–4), and most (highest quintile) deprived. Body mass index (BMI) was grouped into four categories according to WHO guidelines^32^: underweight (BMI < 18.5 kg/m^2^); normal weight (18.5–24.9 kg/m^2^); overweight (25.0–29.9 kg/m^2^); obese (at least 30.0 kg/m^2^). Alcohol consumption was categorized into never, former and current drinker. Family history of dementia is a composite risk factor reflecting both genetic and non-genetic risks. Its relevant effects are separable from that of APOE ε4 allele^33^, and could strengthen the prediction power of the model together with PRS^34^. Thus, family history is incorporated as a covariate in the model.

### Statistical analysis

Cox proportional hazards regression model was used to determine risk of incident dementia during follow-up. Participants who did not develop dementia during the follow-up were censored in this study. Two multiplicative terms *APOE genotype x smoking* and *polygenic risk x smoking* were added into independent Cox models to assess APOE genotype-by-smoking and polygenic risk-by-smoking interactions, respectively. We compared the full model (with the interaction term of *APOE genotype-by-smoking* or *polygenic risk-by-smoking*) with a reduced model (without the interaction term) using a likelihood ratio test. Besides multiplicative interaction, we also evaluated an additive interaction effect by calculating the relative excess risk due to interaction (RERI), attributable proportion (AP) and synergy index (S). All models were adjusted for age, sex, educational attainment, deprivation quintile, BMI, parental family history of dementia and the first five genetic principal components derived from genetic data^35^ for population stratification. Effects were estimated by hazards ratios (HR) with 95% confidence intervals (95% CI). The proportional hazards assumption was checked for each Cox model by measuring Schoenfeld residuals, and results indicated no potential violation of the assumption. We used complete case analysis since the proportion of missing data for main variables was less than 5%. We conducted three sensitivity analyses. First, we stratified the sample by sex and repeated the reduced model and full model. Second, we adjusted for self-reported depression assessed by the two-item Patient Health Questionnaire (PHQ-2)^36^. The range of PHQ-2 score is from 0 to 6 with a score ≥ 3 indicative of self-reported depression. Additionally, we conducted the main analyses after excluding individuals within three years of follow-up to reduce the possibility of reverse causation bias. Although some overlap in the mechanisms leading to dementia would be expected between Caucasians and non-Caucasians, we didn't conduct a sensitivity analysis among non-White participants due to the insufficient cases in UKB. We used R version 4.0.2 and R packages survival version 3.2-7, survminer version 0.4.8, epiR version1.0-15, and ggplot2 version 3.3.2 for analyses, and a two-tailed *P* value < 0.05 was considered statistically significant.

## Results

### Baseline characteristics

193,198 participants were included in the analysis. The mean (SD) age of the participants at baseline was 64.1 (2.9) years, and 101,322 (52.4%) were female. They were followed up for 1,700,886 person-years (median [interquartile range] follow-up, 9.0 [8.3–9.7] years). One thousand seven hundred and eighty-eight cases of incident all-cause dementia were identified. Baseline characteristics of the participants are summarized in Table 1.

**Table 1:** Baseline characteristics of participants.

	No incident dementia (n = 191,410) (%)	Incident dementia (n = 1788) (%)^b^
Age, mean (SD), years	64.1 (2.8)	65.8 (2.7)
**Sex**		
Female	100,525 (52.5)	797 (44.6)
Male	90,885 (47.5)	991 (55.4)
**Educational attainment**		
Higher education	47,905 (25.0)	322 (18.0)
Other qualifications^‡^	141,100 (73.7)	1416 (79.2)
Missing	2405 (1.3)	50 (2.8)
**Deprivation quintile**		
1 (Least deprived)	38,317 (20.0)	317 (17.7)
2–4 (Intermediate deprived)	114,828 (60.0)	969 (54.2)
5 (Most deprived)	38,105 (19.9)	501 (28.0)
Missing	160 (0.1)	1 (0.1)
**Number of APOE ε4 alleles**		
Zero	137,642 (71.9)	817 (45.7)
One	49,458 (25.8)	763 (42.7)
Two	4310 (2.3)	208 (11.6)
**Polygenic risk category**		
Low	38,386 (20.1)	254 (14.2)
Middle	114,861 (60.0)	1057 (59.1)
High	38,163 (19.9)	477 (26.7)
**Smoking status**		
Never	94,979 (49.6)	771 (43.1)
Previous	80,067 (41.8)	818 (45.7)
Current	15,458 (0.1)	185 (10.3)
Missing	906 (0.5)	14 (0.8)
**Alcohol-intake status**		
Never	7933 (4.1)	121 (6.8)
Previous	6973 (3.6)	136 (7.6)
Current	176,340 (92.1)	1526 (85.3)
Missing	164 (0.1)	5 (0.3)
**Body mass index (BMI, kg/m**^**2**^**)**		
Normal	56,088 (29.3)	518 (29.0)
Underweight	809 (0.4)	15 (0.8)
Overweight	86,562 (45.2)	773 (43.2)
Obese	47,272 (24.7)	465 (26.0)
Missing	679 (0.3)	17 (1.0)
**Parents who had dementia**		
None	146,627 (76.6)	1209 (67.6)
Father	7946 (4.2)	89 (5.0)
Mother	19,718 (10.3)	237 (13.3)
Both	1266 (0.7)	20 (1.1)
Missing	15,853 (8.3)	233 (13.0)

^a^Other qualifications indicate without a college or university-level degree.

^b^Percentages may not sum to 100 because of rounding.

### APOE genotype, polygenic risk and dementia risk

Dementia risk was significantly higher among APOE-ε4 allele carriers compared to non-ε4 allele carriers (Supplementary Fig. S2). Specifically, two APOE-ε4 alleles carriers were at greatest risk of dementia followed by one-ε4 allele carriers compared with non-carriers, with an adjusted hazard ratio (HR) of 7.63 (95% confidence interval [CI] 6.46–9.01) and 2.47 (95% CI 2.22–2.76), respectively (Table 2). The strength of the associations was slightly attenuated after additionally adjusting for smoking status with HR of 7.53 (95% CI 6.36–8.91) for two-ε4 allele carriers and 2.45 (95% CI 2.20–2.74) for one-ε4 allele carriers (Supplementary Fig. S3). Individuals with intermediate and high polygenic risk were at greater risk of developing dementia (Supplementary Fig. S2). The adjusted hazard ratio (HR) of dementia was 1.53 (95% CI 1.29–1.81) for individuals with high polygenic risk and 1.28 (95% CI 1.10–1.49) for those with intermediate polygenic risk (Table 2). Strength of the associations between polygenic risk and dementia risk were slightly stronger after additionally controlling for smoking status, with HR of 1.55 (95% CI 1.31–1.83) for high polygenic risk and 1.29 (95% CI 1.11–1.50) for intermediate polygenic risk individuals (Supplementary Fig. S3).

**Table 2:** Risk of incident dementia according to APOE genotype, polygenic risk and smoking status.

	No. of dementia cases/no. of person at risk	HR	95% CI	*P* value
**Model 1**				
Number of APOE ε4 alleles				
Zero	817/138,459	Reference	Reference	Reference
One	763/50,221	2.47	2.22–2.76	< 0.001
Two	208/4518	7.63	6.46–9.01	< 0.001
Polygenic risk category				
Low	254/38,640	Reference	Reference	Reference
Middle	1057/115,918	1.28	1.10–1.49	0.001
High	477/38,640	1.53	1.29–1.81	< 0.001
**Model 2**				
Smoking status				
Never	771/955,750	Reference	Reference	Reference
Previous	818/80,885	1.20	1.07–1.34	0.001
Current	185/15,643	1.36	1.13–1.63	0.001

Model 1 and Model 2 were adjusted for age, sex, educational attainment, deprivation quantile, BMI, alcohol-intake status, parental dementia status and 5 principal components of ancestry, respectively.

*HR* hazard ratio, *CI* confidence interval.

### Smoking status with dementia risk

Dementia risk was significantly associated with smoking (Supplementary Fig. S2; Table 2). Current and former smokers had a higher risk of dementia than those who never smoked (HR 1.36; 95% CI 1.13–1.63) and HR 1.20; 95% CI 1.07–1.34, respectively). The hazards ratio of dementia risk for current and former versus never smokers remained nearly unchanged after the additional adjustment of APOE genotype and polygenic risk (HR 1.37; 95% CI 1.14–1.65 and HR 1.19; 95% CI 1.06–1.32, respectively) (Supplementary Fig. S3).

### Interaction between APOE genotype and smoking on dementia risk

**Figure 1:**
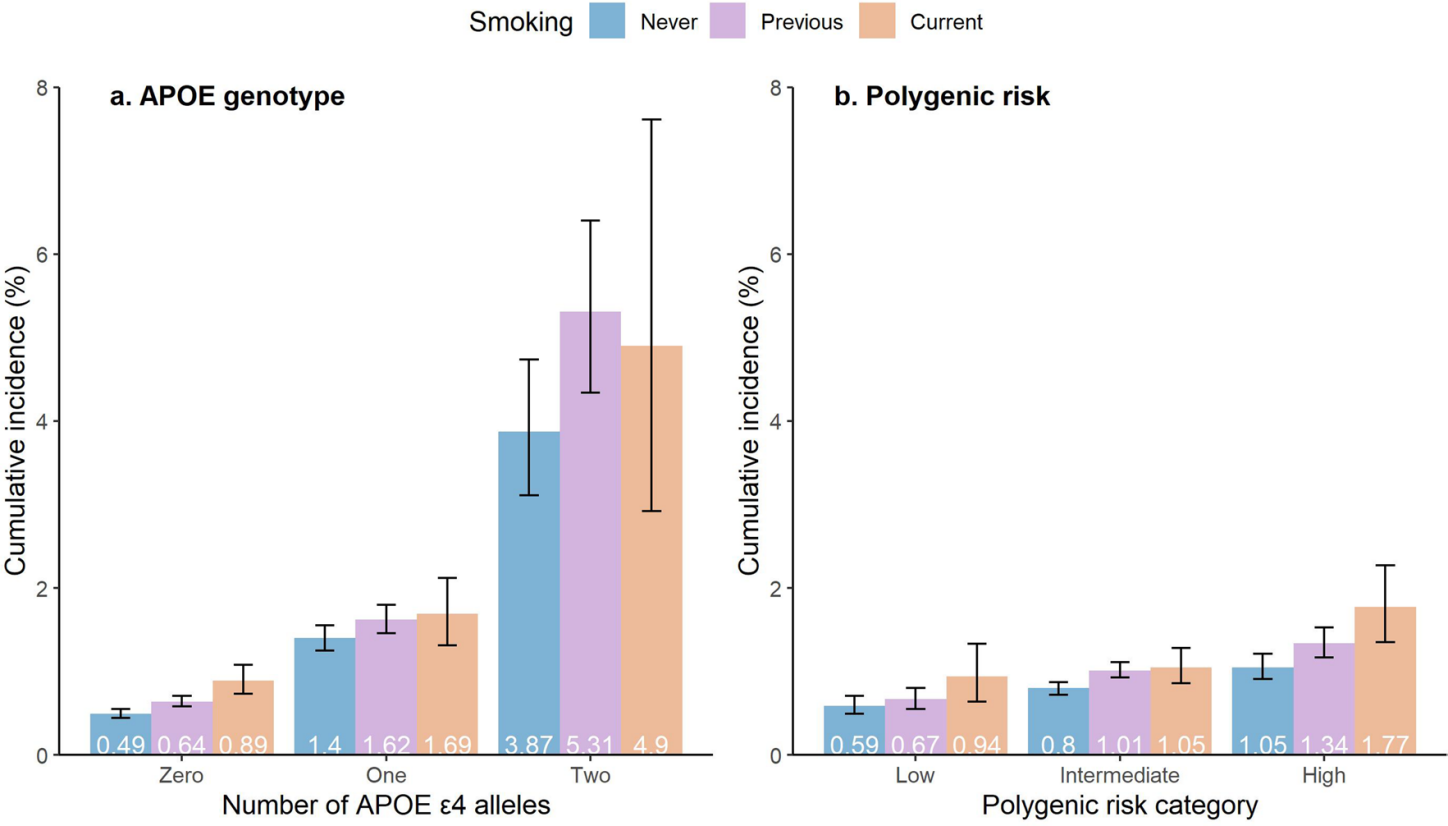
Cumulative incidence of dementia according to genetic predispostion and smoking status. Shown are cumulative incidence of dementia according to APOE genotype, polygenic risk category, and smoking status. The I bars represent 95% confidence intervals.

Cumulative incidence of dementia according to APOE genotype and smoking status is shown in Fig. 1a. Among participants with zero APOE-ε4 allele, 0.89% (95% CI 0.73–1.08%) current and 0.64% (95% CI 0.58–0.71%) former smokers developed dementia compared with 0.49% (95% CI 0.44–0.55%) of never smokers (adjusted HR 1.78; 95% CI 1.39–2.29; *P* = 0.01 and HR 1.23; 95% CI 1.05–1.46; *P* < 0.001, respectively) (Fig. 1a; Table 3). In contrast, among one APOE-ε4 allele carriers, 1.69% (95% CI 1.31–2.12%) current and 1.62% (95% CI 1.46–1.80%) former smokers developed dementia compared with 1.40% (95% CI 1.25–1.55%) of never smokers (adjusted HR 1.06; 95% CI 0.77–1.45; *P* = 0.73 and HR 1.11; 95% CI 0.94–1.32; *P* = 0.21, respectively); of two APOE-ε4 alleles carriers, 4.90% (95% CI 2.92–7.61%) current and 5.31% (95% CI 4.34–6.40%) former smokers developed dementia compared with 3.87% (95% CI 3.11–4.74%) of never smokers (adjusted HR 0.94; 95% CI 0.49–1.79; *P* = 0.84 and HR 1.32; 95% CI 0.96–1.81; *P* = 0.09, respectively) (Fig. 1a; Table 3). There was significant negative multiplicative interaction between current smoking and one APOE-ε4 allele carriers (HR 0.54; 95% CI 0.36–0.80; *P* = 0.002) (Table 4). However, the additive interaction between current smoking and one APOE-ε4 allele carriers was not significant (RERI − 0.85, 95% CI − 1.84–0.13; AP − 0.30, 95% CI − 0.71–0.12; S 0.68, 95% CI 0.42–1.11). Neither multiplicative interaction (HR of 0.53 [95% CI 0.27–1.05]; *P* = 0.07) nor additive interaction (RERI − 0.93, 95% CI − 5.95–4.10; AP − 0.12, 95% CI − 0.82–0.58; S 0.88, 95% CI 0.43–1.81) between current smoking and two APOE-ε4 alleles carriers was significant (Table 4).

**Table 3:** Risk of incident dementia according to smoking status within each APOE genotype and polygenic risk category.

	No. of dementia cases/no. of person at risk	HR	95% CI	*P* value
**Number of APOE ε4 alleles**				
*Zero*				
Smoking status				
Never	339/68,746	Reference	Reference	Reference
Previous	372/57,740	1.23	1.05–1.46	0.01
Current	101/11,321	1.78	1.39–2.29	< 0.001
*One*				
Smoking status				
Never	346/24,781	Reference	Reference	Reference
Previous	344/21,223	1.11	0.94–1.32	0.21
Current	67/3975	1.06	0.77–1.45	0.73
*Two*				
Smoking status				
Never	86/2223	Reference	Reference	Reference
Previous	102/1922	1.32	0.96–1.81	0.09
Current	17/347	0.94	0.49–1.79	0.84
**Polygenic risk category**				
*Low*				
Smoking status				
Never	116/19,552	Reference	Reference	Reference
Previous	106/15,850	1.04	0.78–1.40	0.77
Current	29/3075	1.28	0.79–2.07	0.32
*Middle*				
Smoking status				
Never	456/57,324	Reference	Reference	Reference
Previous	494/48,758	1.23	1.06–1.41	0.01
Current	98/9295	1.25	0.97–1.60	0.09
*High*				
Smoking status				
Never	199/18,874	Reference	Reference	Reference
Previous	218/16,277	1.21	0.97–1.50	0.09
Current	58/3273	1.63	1.16–2.28	0.005

All analyses were adjusted for age, sex, educational attainment, deprivation quantile, BMI, parental dementia status, and 5 principal components of ancestry.

*HR* hazard ratio, *CI* confidence interval.

**Table 4:** Interactions between smoking status and genetic predisposition on dementia risk.

Variables	Additive interaction effect			Multiplicative interaction effect		
	RERI (95% CI)	AP (95% CI)	S (95% CI)	HR (95% CI)	*P* value	*P* value for interaction
Smoking status * number of APOE ε4 alleles	–	–	–	–	–	0.002
Never * zero	–	–	–	Reference	Reference	–
Previous * one	− 0.11 (− 0.63–0.40)	− 0.04 (− 0.21–0.14)	0.95 (0.74–1.21)	0.82 (0.66–1.03)	0.09	–
Current * one	− 0.85 (− 1.84–0.13)	− 0.30 (− 0.71–0.12)	0.68 (0.42–1.11)	0.54 (0.36–0.80)	0.002	–
Previous * two	2.00 (− 0.80–4.81)	0.20 (− 0.05–0.44)	1.28 (0.91–1.79)	1.00 (0.71–1.41)	0.99	–
Current * two	− 0.93 (− 5.95–4.10)	− 0.12 (− 0.82–0.58)	0.88 (0.43–1.81)	0.53 (0.27–1.05)	0.07	–
Smoking status * polygenic risk category	–	–	–	–	–	0.25
Never * low	–	–	–	Reference	Reference	–
Previous * middle	0.24 (− 0.08–0.56)	0.16 (− 0.07–0.39)	2.03 (0.38–10.93)	1.19 (0.87–1.63)	0.29	–
Current * middle	− 0.05 (− 0.77–0.67)	− 0.03 (− 0.51–0.44)	0.91 (0.26–3.24)	0.92 (0.54–1.57)	0.77	–
Previous * high	0.26 (− 0.16–0.68)	0.15 (− 0.09–0.40)	1.58 (0.60–4.16)	1.17 (0.82–1.67)	0.39	–
Current * high	0.49 (− 0.43–1.41)	0.22 (− 0.15–0.58)	1.62 (0.59–4.44)	1.17 (0.66–2.08)	0.58	–

HR, hazard ratio; CI: confidence interval; RERI, relative excess risk due to interaction; AP, attributable proportion; S, synergy index; RERI = 0; AP = 0; S = 1: no interaction; RERI > 0; AP > 0; S > 1: positive interaction; RERI < 0; AP < 0; S < 1: negative interaction.

Dash “–”: not applicable. *P* value for interaction: results from likelihood ratio test to compare the reduced model excluding interaction term with the full model including the term. All analyses were adjusted for age, sex, educational attainment, deprivation quantile, BMI, parental dementia status, and 5 principal components of ancestry.

### Interaction between polygenic risk and smoking status on dementia risk

Cumulative incidence of dementia according to polygenic risk category and smoking status is shown in Fig. 1b. Of individuals with low polygenic risk, 0.94% (95% CI 0.64–1.33%) current and 0.67% (95% CI 0.55–0.80%) former smokers developed dementia compared with 0.59% (95% CI 0.49–0.71%) of never smokers (adjusted HR 1.28; 95% CI 0.79–2.07; *P* = 0.32 and HR 1.04; 95% CI 0.78–1.40; *P* = 0.77, respectively) (Fig. 1b; Table 3). In contrast, of individuals with intermediate polygenic risk, 1.05% (95% CI 0.86–1.28%) current and 1.01% (95% CI 0.93–1.11%) former smokers developed dementia compared with 0.80% (95% CI 0.72–0.87%) of never smokers (adjusted HR 1.25; 95% CI 0.97–1.60; *P* = 0.09 and HR 1.23; 95% CI 1.06–1.41; *P* = 0.01, respectively); and of individuals with high polygenic risk, 1.77% (95% CI 1.35–2.27%) current and 1.34% (95% CI 1.17–1.53%) former smokers developed dementia compared with 1.05% (95% CI 0.91–1.21%) of never smokers (adjusted HR 1.63; 95% CI 1.16–2.28; *P* = 0.005 and HR 1.21; 95% CI 0.97–1.50; *P* = 0.09, respectively) (Fig. 1b; Table 3). Neither multiplicative (HR of 1.17 [95% CI 0.66–2.08]; *P* = 0.58) nor additive interaction (RERI 0.49, 95% CI − 0.43–1.42; AP 0.22, 95% CI − 0.15–0.58; S 1.62, 95% CI 0.59–4.44) between current smoking and high polygenic risk was significant (Table 4). Moreover, there was no significant multiplicative (HR of 0.92 [95% CI 0.54–1.57]; *P* = 0.77) or additive interaction (RERI − 0.05, 95% CI − 0.77–0.67; AP − 0.03, 95% CI − 0.51–0.44; S 0.91, 95% CI 0.26–3.24) between current smoking and intermediate polygenic risk (Table 4).

### Sensitivity analyses

The association between genetic predisposition and dementia risk was similar between men and women (Supplementary Fig. S4). However, current and former smoking were associated with greater dementia risk among women (HR 1.55; 95% CI 1.16–2.07 and HR 1.29; 95% CI 1.10–1.52, respectively) compared with never smokers but not in men (HR 1.25; 95% CI 0.98–1.59 and HR 1.10; 95% CI 0.95–1.28, respectively). There was significant multiplicative interaction between smoking status and APOE genotype among women (interaction *P* = 0.01) while no additive interaction was detected (Supplementary Table S1). Neither multiplicative interaction nor additive interaction between smoking status and polygenic risk was observed on dementia risk among women. For men, there was significant additive interaction (RERI 3.81, 95% CI 0.67–6.96; AP 0.40, 95% CI 0.14–0.66; S 1.81, 95% CI 1.09–3.01) between one APOE-ε4 allele carriers and former smoking on dementia risk while non-significant multiplicative interaction was identified (HR 1.49; 95% CI 0.92–2.41) (Supplementary Table S2). The interaction between smoking and genetic predisposition was similar with the main analyses after additionally adjusting for self-reported depression (Supplementary Table S3) and after excluding participants followed up for less than three years (Supplementary Table S4).

## Discussion

To our knowledge, this study is the first large population-based analysis exploring the interaction between APOE, polygenic risk and smoking in relation to dementia risk. APOE genotype modified the association between smoking and dementia risk. However, there was no interactive effect of current or past smoking with polygenic risk on the risk of dementia. Carriers of two ε4 alleles (APOE ε4ε4 genotype) had the greatest risk of developing dementia after adjusting for potential confounders. APOE-ε4 allele is the strongest genetic risk for late-onset form of Alzheimer’s disease (LOAD). Individuals with higher polygenic risk had a greater dementia risk indicative of the polygenic architecture of dementia^14^.

Tobacco usage may worsen cognitive function and increase risk of dementia^23,37,38^. Smoking may induce cerebral oxidative stress that accelerate Alzheimer disease pathology and increase its risk^39^. In this large population-based study, both current and previous smokers were at increased risk of dementia. Conversely, neither current nor former smokers had higher dementia risk among carriers of one or two ε4 alleles. Our study indicated that APOE-ε4 genotype was the strongest risk for dementia in the model adjusting for genetic predisposition and smoking. It increased dementia risk in a way such that the relative risk of other risk factors including smoking on dementia weakened in APOE ε4 heterozygotes, and even disappeared in ε4 homozygotes. APOE genotype modified the association between smoking and dementia risk. APOE-ε4 allele carriers who were also smokers demonstrated greater cortical amyloid deposition, poor auditory-verbal learning and memory^40^ which might increase the risk of developing dementia. Our findings were in agreement with the results of two Rotterdam studies^22,23^ that the magnitude of risk elevation seen between current smokers and never smokers was greatest among non-carriers of the ε4 allele. However, the later Rotterdam study in 2007 concluded that there was no interaction between smoking status and APOE genotype on risk of dementia^23^. One possible explanation for this inconsistency may lie in the larger sample size in our study which increases statistical power to detect interactions. Our study demonstrated current smokers within the highest polygenic risk quintile had an increased risk of developing dementia compared to never smokers, whereas there was no significant association between current smoking and dementia risk among individuals at lower or intermediate polygenic risk. Consistent with a previous study that reported non-significant interaction between lifestyle and polygenic risk on dementia risk^16^, we also found no significant multiplicative or additive interaction between smoking status and polygenic risk. Our sensitivity analyses showed that current and past smoking increased the dementia risk only among women, after adjusting for genetic predisposition and other covariates. Female smokers are more vulnerable to cardiovascular disease^41^ which further increases their risk of dementia. APOE genotype modified the effect of smoking status on dementia risk among women and men in different ways. Current smoking and ε4 heterozygotes had significant negative multiplicative interaction while non-significant additive interaction among women. However, among men, past smoking and ε4 homozygotes had significant additive interaction while non-significant multiplicative interaction among men. It is possible that smoking status and APOE genotype have a negative multiplicative interaction while non-significant additive interaction or a significant positive additive interaction effect while non-significant multiplicative interaction since the two effects depend on different scale. The additive interaction is based on a risk difference scale with a larger effect while the multiplicative interaction is based on a risk ratio scale with a relatively smaller effect^42^.

The strengths of this study include the large population-based sample, the long follow-up period, the comprehensive approach to investigating genetic risk, the novel investigation of interactions, the careful adjustment for potential confounders, and the use of sensitivity analyses to investigate the robustness of findings.

Our study also had a number of limitations: First, the algorithmically-defined dementia cases including both primary care information and hospital or death registry linked data used in this study is likely to include misclassified or misdiagnosed participants. However, the algorithm was developed to balance sensitivity and specificity^29^. Incorporating primary care data may reduce the proportion of missed dementia cases or false negatives in health or death registry records. Second, data on smoking status was addressed only once, at baseline, which would not capture change of these health behaviors during the follow-up. Third, although the models were adjusted for known potential confounders, the possibility of residual or unmeasured confounders may affect the results. Fourth, our study sample included individuals aged between 60 and 65 years old. The majority of UK Biobank participants over the age of 60 are younger than 65, and comparatively healthier than the general population^43^. Over 9 years follow-up, there were 1788 (0.93%) incident cases in this younger group compared to 1231 (1.4%) incident cases among those aged greater than 65 years. Fifth, our polygenic risk score (PRS) is made for AD, but the outcome is any type of dementia. However, PRS for AD could predict clinical diagnosis of all-cause dementia^17,44^. Lastly, the participants in this study was restricted to older White British adults that might limit the generalizability of the findings to other ethnicities.

In conclusion, this study demonstrates how genetic predisposition modifies the association between smoking status and dementia risk. Current smokers had a higher risk of dementia among non-APOE-ε4 allele carriers and among individuals within high polygenic risk. For smokers among APOE-ε4 allele carriers, the prevention strategies on dementia might be more effective by alleviating the negative effect of APOE ε4 allele and smoking via other composite measures rather than solely through smoking control.

## Data availability

The data that support the findings of this study are available from the corresponding author on reasonable request.

## Supplementary Information

Supplementary Informations.
