# Supplementary material for: Interaction between genetic predisposition, smoking, and dementia risk: a population-based cohort study
Source: Sci Rep. 2021 Jun 21;11:12953. doi: 10.1038/s41598-021-92304-x (PMC8217565; doi:10.1038/s41598-021-92304-x)
Supplement: Supplementary file 1 — Supplementary Informations. [file 41598_2021_92304_MOESM1_ESM.pdf]

SUPPLEMENTARY INFORMATION

Interaction between genetic predisposition, smoking, and dementia risk: a population-based cohort study

Na Zhang[1], Janice M. Ranson[2], Zhi-Jie Zheng[1], Eilis Hannon[2], Zhenwei Zhou[3], Xuejun Kong[4,5], David J. Llewellyn[2,6], Daniel A. King[7], Jie Huang[1*]

[1]Department of Global Health, Peking University School of Public Health, Beijing, China;

[2]College of Medicine and Health, University of Exeter, Exeter, UK

[3]Department of Biostatistics, Boston University, Boston, MA, USA

[4]Department of Psychiatry, Beth Israel Deaconess Medical Center, Boston, MA, USA

[5]Martinos Center, Massachusetts General Hospital, Charlestown, MA, USA

[6]Alan Turing Institute, London, UK

[7]Department of Medicine, Stanford University School of Medicine, Stanford, CA, USA

[*]**Corresponding author:**

Jie Huang

E-mail: jiehuang001@pku.edu.cn

Tel: (86) 152 1008 1889

Supplementary Table S1. Interaction between smoking status and genetic predisposition on dementia risk among females

Supplementary Table S2. Interaction between smoking status and genetic predisposition on dementia risk among males

Supplementary Table S3. Interaction between smoking status and genetic predisposition on dementia risk additionally adjusted for depression status

Supplementary Table S4. Interaction between smoking status and genetic predisposition on dementia risk (excluded samples followed up less than 3 years)

Supplementary Figure S1. Flow chart of the study population

Supplementary Figure S2. Cumulative incidence of dementia in groups stratified by a. APOE-related risk, b. polygenic risk score category, and c. smoking status during follow-up years

Supplementary Figure S3. Adjusted hazard ratio of APOE genotype, polygenic risk, smoking status on dementia risk

Supplementary Figure S4. Association of genetic predisposition and smoking status with dementia risk by sex

Supplementary Figure S1. Flow chart of the study population

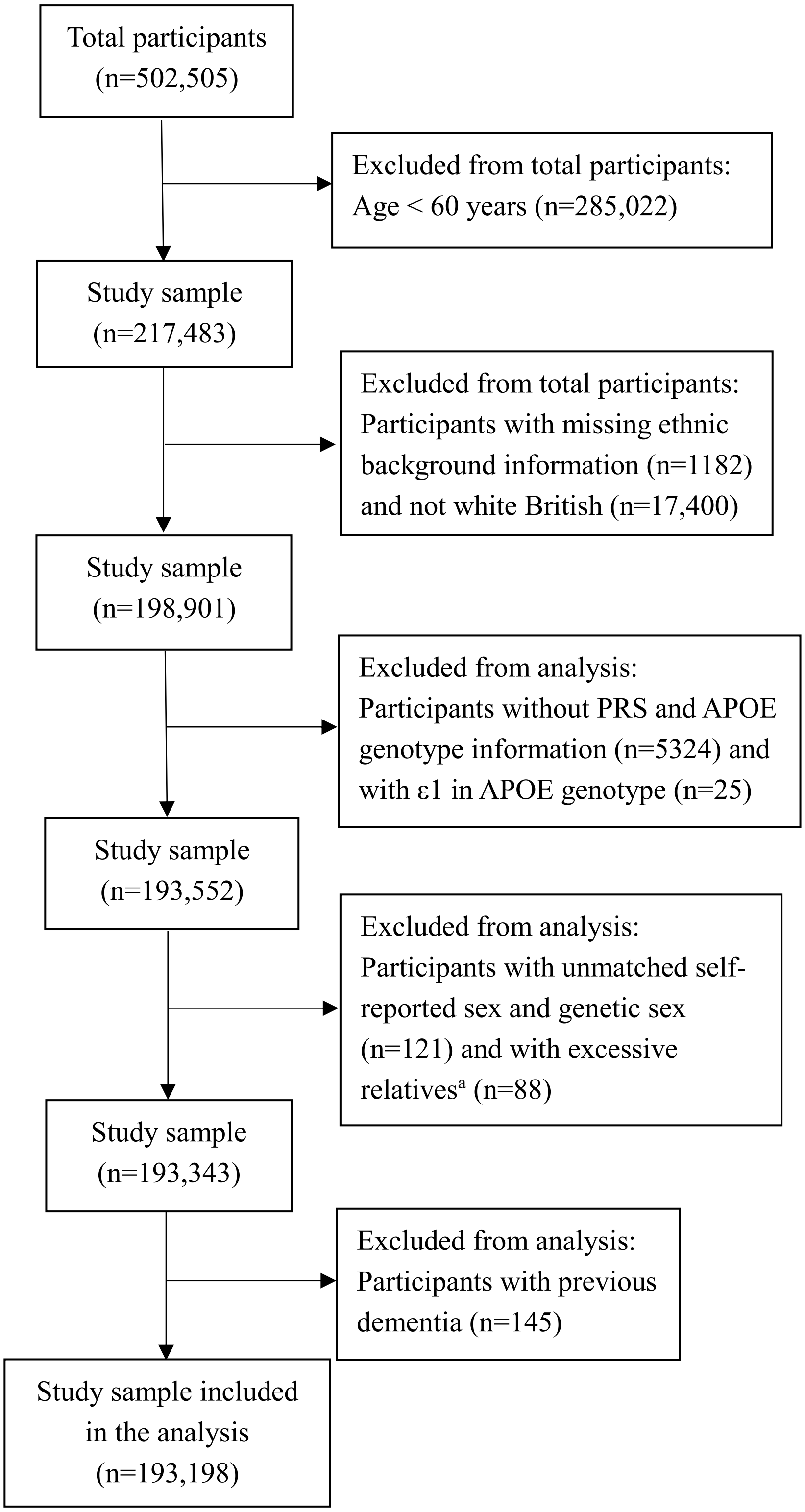

[a]Excessive relatives: more than 10 putative third-degree relatives.

Supplementary Figure S2. Cumulative incidence of dementia in groups stratified by a. APOE-related risk, b. polygenic risk score category, and c. smoking status during follow-up years

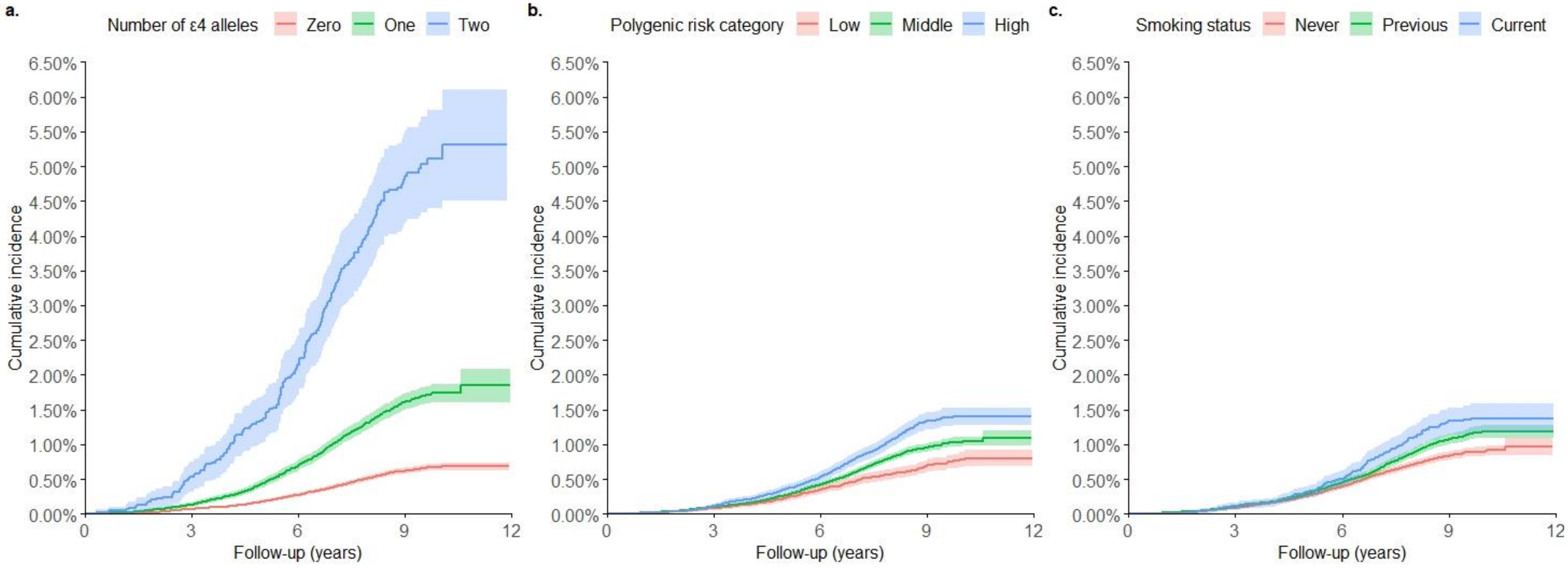

Supplementary Figure S3. Adjusted hazard ratio of APOE genotype, polygenic risk, smoking status on dementia risk

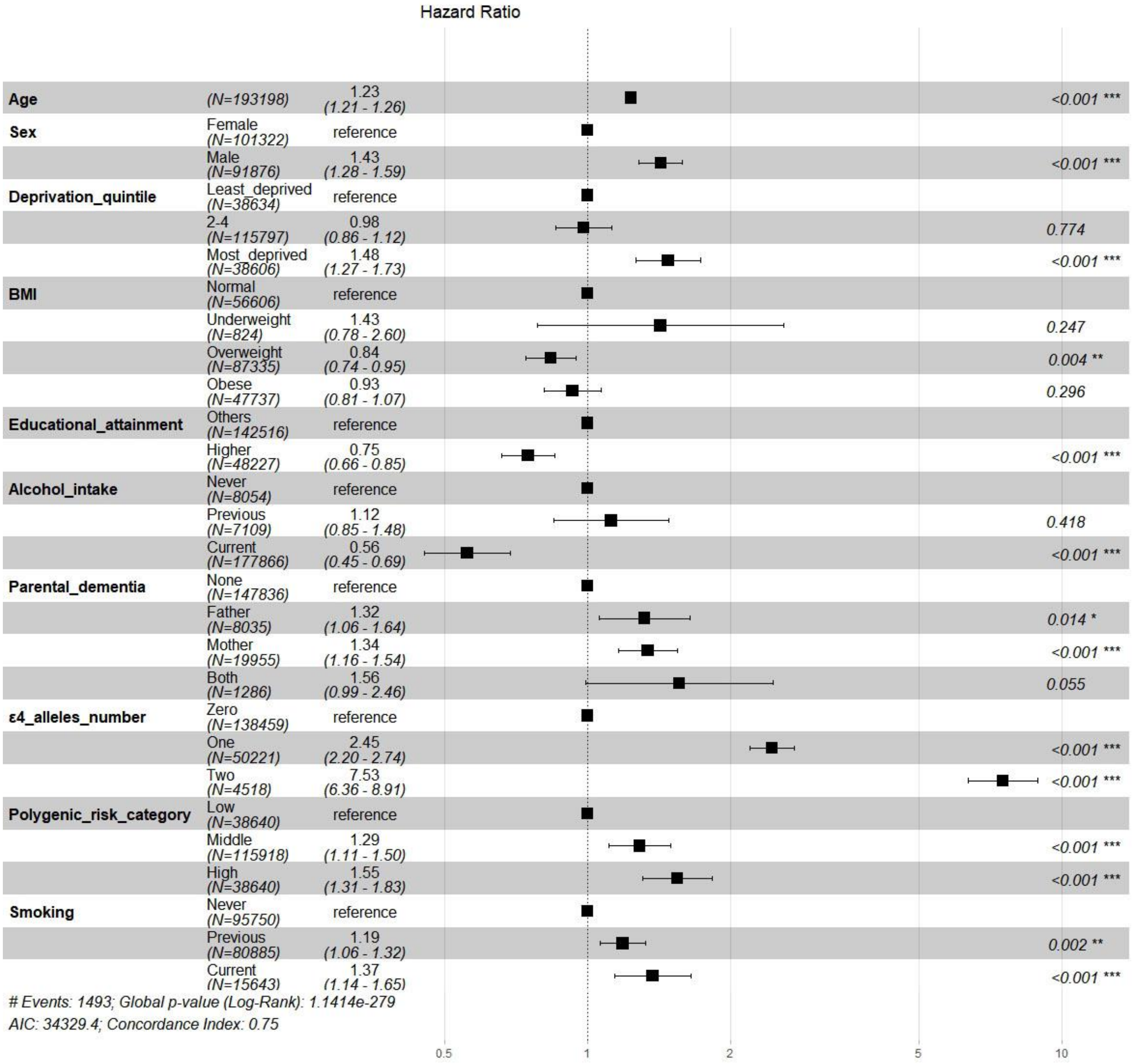

*: *P*<0.05; **: *P*<0.01; ***: *P*<0.001.

Adjusted for 5 principal components of ancestry.

Supplementary Figure S4. Association of genetic predisposition and smoking status with dementia risk by sex

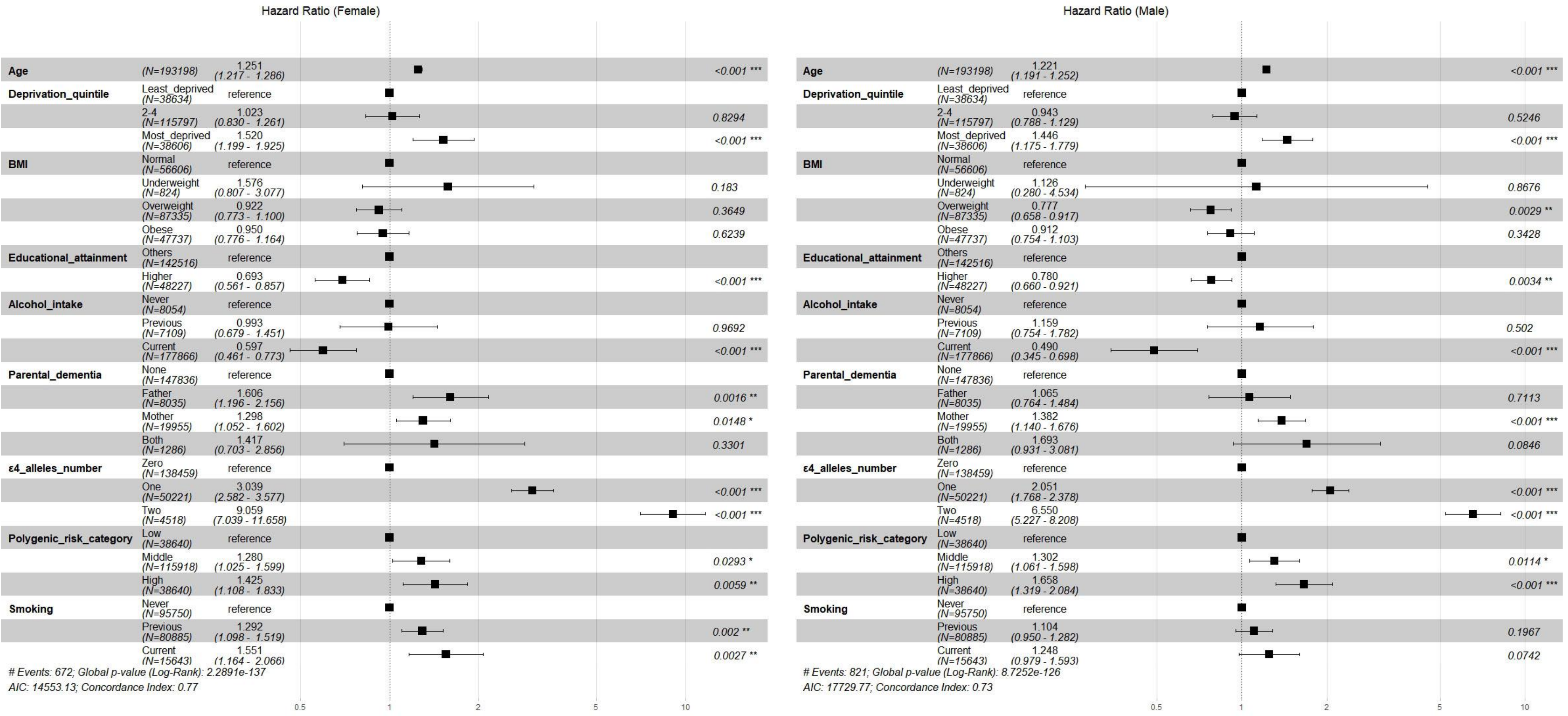

*: *P*<0.05; **: *P*<0.01; ***: *P*<0.001.

Both models adjusted for 5 principal components of ancestry.

Supplementary Table S1. Interaction between smoking status and genetic predisposition on dementia risk among females

Variables	Additive interaction effect			Multiplicative interaction effect		
	RERI (95% CI)	AP (95% CI)	S (95% CI)	HR (95% CI)	*P* value	*P* value for interaction
Smoking status * Number of APOE ε4 alleles	-	-	-	-	-	0.01
Never * Zero	-	-	-	Reference	-	-
Previous * One	0.60 (-0.34-1.55)	0.14 (-0.06-0.34)	1.22 (0.89-1.67)	0.92 (0.66-1.30)	0.64	-
Current * One	-1.19 (-3.06-0.67)	-0.36 (-1.07-0.35)	0.66 (0.32-1.37)	0.46 (0.24-0.86)	0.02	
Previous * Two	-1.47 (-6.49-3.55)	-0.15 (-0.69-0.4)	0.86 (0.51-1.46)	0.64 (0.37-1.10)	0.11	-
Current * Two	6.12 (-7.11-19.36)	0.33 (-0.16-0.83)	1.55 (0.7-3.42)	0.77 (0.33-1.79)	0.54	-
Smoking status * Polygenetic risk category	-	-	-			0.15
Never * Low	-	-	-	Reference		-
Previous * Middle	0.44 (-0.04-0.92)	0.27 (-0.04-0.58)	3.30 (0.14-75.39)	1.36 (0.85-2.19)	0.20	-
Current * Middle	-0.16 (-1.41-1.09)	-0.10 (-0.90-0.70)	0.79 (0.14-4.54)	0.87 (0.39-1.94)	0.73	-
Previous * High	0.16 (-0.48-0.79)	0.10 (-0.30-0.50)	1.39 (0.29-6.6)	1.10 (0.64-1.88)	0.73	-
Current * High	0.50 (1.04-2.04)	0.20 (-0.37-0.78)	1.52 (0.38-6.17)	1.13 (0.48-2.66)	0.78	-

Abbreviation: HR, hazard ratio; CI: confidence interval; RERI, relative excess risk due to interaction; AP, attributable proportion; S, synergy index; RERI=0; AP=0; S=1: no interaction; RERI>0; AP>0; S>1: positive interaction; RERI<0; AP<0; S<1: negative interaction. Dash "-": not applicable. *P* value for interaction: results from likelihood ratio test to compare the reduced model excluding interaction term with the full model including the term.

All analyses were adjusted for age, sex, educational attainment, deprivation quantile, BMI, parental dementia status, and 5 principal components of ancestry.

Supplementary Table S2. Interaction between smoking status and genetic predisposition on dementia risk among males

Variables	Additive interaction effect			Multiplicative interaction effect		
	RERI (95% CI)	AP (95% CI)	S (95% CI)	HR (95% CI)	*P* value	*P* value for interaction
Smoking status * Number of APOE ε4 alleles	-	-	-	-	-	0.001
Never * Zero	-	-	-	Reference	-	-
Previous * One	-0.35 (-0.96-0.25)	-0.16 (-0.44, 0.12)	0.77 (0.51-1.17)	0.80 (0.58-1.09)	0.15	-
Current * One	-0.57 (-1.65-0.51)	-0.23 (-0.74-0.28)	0.72 (0.36-1.41)	0.64 (0.38-1.05)	0.07	-
Previous * Two	3.81 (0.67-6.96)	0.40 (0.14-0.66)	1.81 (1.09-3.01)	1.49 (0.92-2.41)	0.11	
Current * Two	-3.19 (-7.18-0.80)	-1.08 (-3.53-1.37)	0.38 (0.07-2.2)	0.34 (0.10-1.15)	0.08	-
Smoking status * Polygenetic risk category	-	-	-	-	0.60	0.60
Never * Low	-	-	-	Reference	-	-
Previous * Middle	0.09 (-0.36-0.54)	0.06 (-0.27-0.4)	1.32 (0.23-7.66)	1.07 (0.69-1.64)	0.77	-
Current * Middle	-0.02 (-0.90-0.86)	-0.01 (-0.61-0.58)	0.96 (0.16-5.70)	0.95 (0.47-1.93)	0.46	-
Previous * High	0.3 (-0.27-0.88)	0.17 (-0.15-0.49)	1.61 (0.47-5.54)	1.20 (0.74-1.94)	0.89	-
Current * High	0.46 (-0.7-1.61)	0.21 (-0.28-0.7)	1.64 (0.4-6.76)	1.19 (0.55-2.59)	0.66	-

Abbreviation: HR, hazard ratio; CI: confidence interval; RERI, relative excess risk due to interaction; AP, attributable proportion; S, synergy index; RERI=0; AP=0; S=1: no interaction; RERI>0; AP>0; S>1: positive interaction; RERI<0; AP<0; S<1: negative interaction. Dash "-": not applicable. *P* value for interaction: results from likelihood ratio test to compare the reduced model excluding interaction term with the full model including the term.

All analyses were adjusted for age, sex, educational attainment, deprivation quantile, BMI, parental dementia status, and 5 principal components of ancestry.

Supplementary Table S3. Interaction between smoking status and genetic predisposition on dementia risk additionally adjusted for depression status

Variables	Additive interaction effect			Multiplicative interaction effect		
	RERI (95% CI)	AP (95% CI)	S (95% CI)	HR (95% CI)	*P* value	*P* value for interaction
Smoking status * Number of APOE ε4 alleles	-	-	-	-	-	0.01
Never * Zero	-	-	-	Reference	Reference	-
Previous * One	-0.12 (-0.67-0.44)	-0.04 (-0.21-0.14)	0.95 (0.74-1.21)	0.79 (0.62-1.00)	0.05	-
Current * One	-1.02 (-2.03-0.00)	-0.37 (-0.83-0.09)	0.63 (0.38-1.06)	0.51 (0.34-0.77)	0.001	-
Previous * Two	1.48 (-1.56-4.52)	0.14 (-0.12-0.40)	1.18 (0.84-1.67)	0.88 (0.62-1.25)	0.47	-
Current * Two	-2.06 (-7.18-3.06)	-0.27 (-1.11-0.56)	0.76 (0.36-1.62)	0.47 (0.23-0.95)	0.04	-
Smoking status * Polygenetic risk category	-	-	-	-	-	0.84
Never * Low	-	-	-	Reference	Reference	-
Previous * Middle	0.22 (-0.13-0.56)	0.14 (-0.09-0.38)	1.69 (0.48-5.97)	1.15 (0.83-1.59)	0.41	-
Current * Middle	-0.1 (-0.85-0.65)	-0.07 (-0.57-0.43)	0.84 (0.24-2.92)	0.89 (0.51-1.54)	0.67	-
Previous * High	0.26 (-0.19-0.71)	0.14 (-0.1-0.39)	1.47 (0.64-3.39)	1.14 (0.79-1.64)	0.49	-
Current * High	0.23 (-0.71-1.17)	0.11 (-0.32-0.54)	1.27 (0.46-3.51)	1.02 (0.56-1.86)	0.94	-

Abbreviation: HR, hazard ratio; CI: confidence interval; RERI, relative excess risk due to interaction; AP, attributable proportion; S, synergy index; RERI=0; AP=0; S=1: no interaction; RERI>0; AP>0; S>1: positive interaction; RERI<0; AP<0; S<1: negative interaction. Dash "-": not applicable. *P* value for interaction: results from likelihood ratio test to compare the reduced model excluding interaction term with the full model including the term.

All analyses were adjusted for age, sex, educational attainment, deprivation quantile, BMI, parental dementia status, and 5 principal components of ancestry.

Supplementary Table S4. Interaction between smoking status and genetic predisposition on dementia risk (excluded samples followed up less than 3 years)

Variables	Additive interaction effect			Multiplicative interaction effect		
	RERI (95% CI)	AP (95% CI)	S (95% CI)	HR (95% CI)	*P* value	*P* value for interaction
Smoking status * Number of APOE ε4 alleles	-	-	-	-	-	0.01
Never * Zero	-	-	-	Reference	Reference	-
Previous * One	-0.09 (-0.64-0.47)	-0.03 (-0.21-0.15)	0.96 (0.74-1.24)	0.83 (0.65-1.05)	0.12	-
Current * One	-0.65 (-1.74-0.43)	-0.21 (-0.6-0.19)	0.77 (0.48-1.23)	0.57 (0.38-0.86)	0.01	-
Previous * Two	1.00 (-1.94-3.95)	0.10 (-0.18-0.39)	1.13 (0.79-1.62)	0.89 (0.62-1.28)	0.53	-
Current * Two	-2.03 (-7.18-3.13)	-0.28 (-1.16-0.60)	0.75 (0.34-1.67)	0.46 (0.22-0.96)	0.04	-
Smoking status * Polygenetic risk category	-	-	-	-	-	0.13
Never * Low	-	-	-	Reference	Reference	-
Previous * Middle	0.29 (-0.04-0.62)	0.2 (-0.04-0.44)	2.76 (0.21-36.6)	1.25 (0.89-1.75)	0.19	-
Current * Middle	-0.05 (-0.8-0.71)	-0.03 (-0.53-0.47)	0.92 (0.23-3.62)	0.93 (0.53-1.62)	0.80	-
Previous * High	0.28 (-0.16-0.72)	0.16 (-0.09-0.42)	1.65 (0.57-4.78)	1.20 (0.83-1.75)	0.33	-
Current * High	0.65 (-0.34-1.64)	0.26 (-0.09-0.62)	1.79 (0.65-4.92)	1.24 (0.68-2.26)	0.48	-

Abbreviation: HR, hazard ratio; CI: confidence interval; RERI, relative excess risk due to interaction; AP, attributable proportion; S, synergy index; RERI=0; AP=0; S=1: no interaction; RERI>0; AP>0; S>1: positive interaction; RERI<0; AP<0; S<1: negative interaction. Dash "-": not applicable. *P* value for interaction: results from likelihood ratio test to compare the reduced model excluding interaction term with the full model including the term.

All analyses were adjusted for age, sex, educational attainment, deprivation quantile, BMI, parental dementia status, and 5 principal components of ancestry.
